# Better oral hygiene is associated with a reduced risk of cataract: A nationwide cohort study

**DOI:** 10.3389/fmed.2022.1036785

**Published:** 2023-01-03

**Authors:** Jung-Hyun Park, Heajung Lee, Jin-Woo Kim, Tae-Jin Song

**Affiliations:** ^1^Department of Oral and Maxillofacial Surgery, Mokdong Hospital, College of Medicine, Ewha Womans University, Seoul, South Korea; ^2^Department of Neurology, Seoul Hospital, College of Medicine, Ewha Womans University, Seoul, South Korea

**Keywords:** periodontitis, oral hygiene, cataract, epidemiology, oral inflammation

## Abstract

**Objective:**

To investigate the association of oral health status and oral hygiene behaviors with cataract occurrence longitudinally.

**Materials and methods:**

Based on the National Health Screening cohort database of Korea, participants who underwent oral health screening by dentists in 2003 were included. Cataract was defined as two or more claims of disease classification for the International Classification of Diseases-10 (E10.34, E11.34, E12.34, E13.34, E14.34, H25, and H26) with cataract specific treatment or surgery procedure claim codes. The occurrence of cataract was analyzed with Cox proportional hazard model according to the presence of periodontitis and oral health examination findings, including missing teeth, caries, tooth brushing, and dental scaling.

**Results:**

Overall, 103,619 subjects were included. During a median follow-up of 12.2 years, cataract developed in 12,114 (11.7%) participants. Poor oral health status such as the presence of periodontitis (adjusted hazard ratio [HR] 1.08, 95% CI [confidence interval] 0.99–1.17, *p* = 0.088) and increased number of missing teeth (adjusted HR = 1.74, 95% CI = 1.55–1.96, *p* < 0.001) was associated with the increased cataract risk. Better oral hygiene behaviors such as increased frequency of tooth brushing (adjusted HR = 0.84, 95% CI = 0.79–0.88, *p* < 0.001) and performed dental scaling within 1 year (adjusted HR = 0.90, 95% CI = 0.86–0.94, *p* < 0.001) were negatively associated with cataract occurrence.

**Conclusion:**

Periodontitis and increased number of missing teeth may increase the risk of cataract. However, maintaining good oral hygiene through tooth brushing and dental scaling may reduce the risk of future cataract occurrence. Further studies should be performed to confirm the association between chronic oral inflammation and cataract.

## Introduction

Poor oral health conditions such as periodontitis, dental caries, or multiple tooth loss are common conditions in humans (1, 2). Poor oral health not only adversely affects oral health itself but also shows a systematic association with or triggers the occurrence of various diseases (3, 4). For example, periodontitis not only causes local inflammation in the oral cavity but also triggers systemic inflammatory consequences. Furthermore, missing tooth and caries are associated with the risk of various systemic diseases, such as diabetes, cardiovascular disease, certain cancers, and neurodegenerative diseases (1, 4–8).

Cataract is a cloudy area of the crystalline lens inside the eye, leading to a decrease in vision. Cataracts affect approximately 95 million people worldwide and remain the major cause of blindness in middle-income and low-income countries (9). The potential risk factors for cataract are aging, dyslipidemia, diabetes mellitus, and hypertension (10). Inflammatory reaction and cascade play an important role in cataract development at a cellular level (9, 11). Because oral health conditions such as periodontitis, tooth loss, and dental caries are closely related to this systemic inflammation, oral health may be linked to cataract occurrence (12–14). Moreover, a decreased inflammatory reaction following good oral hygiene or behavior can reduce the risk of cataract occurrence (12). However, few related studies have described the association of oral health status with cataract.

We hypothesized that poor oral health would be related to the increased risk of cataract occurrence, and better oral hygiene behavior would be related with a reduced risk of cataract occurrence. Therefore, this study aimed to investigate the association of oral health examination estimates with cataract development longitudinally in a nationwide cohort database.

## Materials and methods

### Data source

The National Health Insurance Service-National Health Screening (NHIS-HEALS) cohort database of Korea was used in this study. The NHIS is administered and supported by the Korean government. As the sole insurance provider in Korea, it covers nearly 97% of Koreans. The remaining 3% of the population is supported by the Medical Aid program, which is administered by the NHIS (15). Subscribers of the NHIS are recommended to receive standardized health screening every 1–2 years. The NIHS-HEALS cohort consists of a database of approximately 510,000 random individuals aged 40–79 years and who participated in health screenings, representing 10% of the total population (16). The cohort database contains individual health screening information, including weight, height, blood pressure, and laboratory tests. It also includes the claims database of diagnosis, prescription, and treatment as well as demographic and socioeconomic information. During health screening, questionnaires on lifestyle, which include oral hygiene behaviors such as frequency of tooth brushing or dental visit within a year, are collected. It also includes oral health examination by a dentist, who examined dental problems such as the number of dental caries or missing teeth. This study was approved by the human subjects ethics board of Ewha Womans University College of Medicine (2020-08-018).

### Study population

**FIGURE 1:**
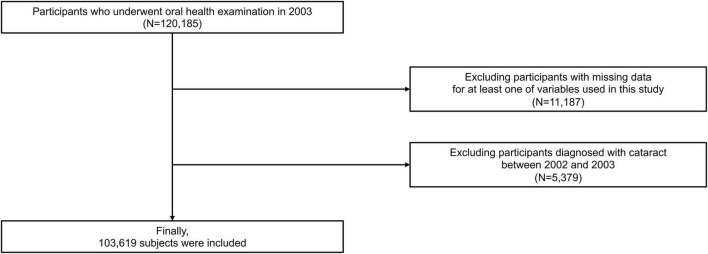
Flow chart of study participants.

From the NHIS-HEALS database, subjects who underwent oral health screening in 2003 were included (*n* = 120,185). Participants (*n* = 11,187) were excluded when data on at least one variable of interest were missing. Participants (*n* = 5,379) with a history of cataract (International Classification of Diseases (ICD)-10 codes E10.34, E11.34, E12.34, E13.34, E14.34, H25, and H26) between January 2002 and December 2003 were excluded. Thus, 103,619 participants were analyzed in this study (Figure 1).

### Definition and variables

The date of oral health examination was set as the index date. The following baseline characteristics on the index date were collected: age, sex, household income, and body mass index. Information on smoking habits (non-smoker, former smoker, and current smoker), alcohol consumption (frequency per week), and regular physical exercise (frequency per week) was obtained using questionnaires. Comorbidities were identified via the following criteria: between January 2002 and index date. Hypertension was defined in patients when they satisfied one of the following criteria: (1) at least one claim of diagnostic codes (ICD-10 I10–15) with the prescription of an antihypertensive agent, (2) two or more claims of diagnostic codes (ICD-10 I10–15), (3) systolic/diastolic blood pressure ≥140/90 mmHg, or 4) self-reported hypertension in the questionnaire. Diabetes mellitus was defined in patients when they met one of the following criteria: (1) at least one claim of diagnostic codes (ICD-10 E11–14) with the prescription of an antidiabetic agent, (2) two or more claims of diagnostic codes (ICD-10 E11–14), (3) fasting serum glucose level ≥7.0 mmol/L, or (4) self-reported diabetes mellitus in the questionnaire. Dyslipidemia was defined when patients satisfied one of the following criteria: (1) at least one claim of diagnostic codes (ICD-10 E78) with the prescription of a dyslipidemia-related agent, (2) two or more claims of diagnostic codes (ICD-10 E78), and (3) total cholesterol ≥240 mg/dl. Atrial fibrillation was defined as two or more claims of diagnostic code (ICD-10 I48). Renal disease was defined as two or more claims of diagnostic codes (ICD-10 N17-19, I12-13, E082, E102, E112, and E132) or estimated glomerular filtration rate of less than 60 ml/min/1.73 m^2^.

The presence of periodontitis was defined according to the following criteria between January 2002 and the index date: (1) two or more claims of ICD-10 codes K052–054 (acute periodontitis [K052], chronic periodontitis [K053], and periodontitis [K054]) with at least one claim of related treatment codes (Supplementary Table 1) or (2) positive checking of a periodontal pocket by a dentist upon oral health examination. The number of missing tooth and dental caries was assessed by a dentist upon oral health examination. The number of dental caries was classified as 0, 1–5, and ≥6 and the number of missing teeth was categorized as 0, 1–7, 8–14, and ≥15 without considering the cause (1, 17). Oral hygiene behaviors were collected as self-reported data during oral health examination: the frequency of tooth brushing per day, dental visit for any reason within the previous year, and dental scaling within the previous year were included.

### Study outcomes

The main outcome was cataract occurrence. Cataract was defined as two or more claims of disease classification for ICD-10 codes (E10.34, E11.34, E12.34, E13.34, E14.34, H25, and H26) with cataract-specific treatment or surgical procedure claim codes. The Korean Electronic Data Interchange codes for cataract treatment were (1) phacoemulsification (S5119), (2) extracapsular or intracapsular cataract extraction (S5111), (3) secondary intraocular lens implantation (S5116), and (4) primary intraocular lens implantation (S5117). Subjects with at least one claim of the following claim codes was not considered as cataract development to exclude false-positive cases: (1) retained magnetic intraocular foreign body (H446), (2) retained nonmagnetic intraocular foreign body (H447), (3) trabeculectomy (S5043), (4) microscopic trabeculotomy (S5047), (5) glaucoma implant insertion (S5049), (6) limited vitrectomy (S5122), and (7) complete vitrectomy (S5121). The follow-up period was from the index date to the occurrence of cataract, the death of a subject, or December 2015, whichever occurred first.

### Statistical analysis

A chi-square test and an independent *t*-test were, respectively, used for categorical and continuous variables to compare the baseline characteristics of groups. Categorical and continuous variables were expressed as numbers (percentages) and means ± standard deviations (SDs), respectively. Kaplan–Meier survival curves were used to evaluate the association of oral health status and oral hygiene behaviors with the incident cataract risk, and a log-rank test was performed to compare the survival curves. The number of cataract cases was divided by the sum of person-years to estimate cataract incidence. Cox’s proportional hazard regression was performed to determine the hazard ratio (HR) with 95% confidence interval (CI) to determine the risk for cataract development in relation to oral health status and oral hygiene behaviors. A multivariable regression model was constructed with adjustment for age, sex, body mass index, household income, alcohol consumption, smoking status, regular physical activity, comorbidities (hypertension, diabetes mellitus, dyslipidemia, atrial fibrillation, and renal disease). The oral health parameters were adjusted in multivariable analysis separately because of multicollinearity. Schoenfeld’s residuals were used to test the assumption of the proportionality of hazards. No violation of the proportional hazard assumption was observed. Data were statistically analyzed using the SAS software (version 9.2, SAS Institute, Cary, NC), and all values were considered statistically significant when *p* < 0.05.

## Results

Among 103,619 participants, the included participants had an average age of 51.64 ± 8.74 years, and 60.1% of them were male. Among them, 1,092 (1.1%) participants had more than 15 missing teeth, 976 (0.9%) participants had 6 or more caries, and 33,439 (32.3%) participants brushed their teeth more than thrice a day (Table 1). Comparative analysis of the baseline characteristics is shown in Supplementary Table 2.

**TABLE 1:** Baseline characteristics of the study population.

	Total
*No. of patients (%)*	1,03,619
*Age*	
Mean (year)	51.64 ± 8.74
*Sex*	
Male	62,248 (60.1)
Female	41,371 (39.9)
*Body mass index (kg/m* ^2^ *)*	23.94 ± 2.91
*Household income*	
T1, lowest	30,353 (29.3)
T2	38,001 (36.7)
T3, highest	35,265 (34.0)
*Alcohol consumption (per week)*	
None	72,370 (69.8)
1–4	27,119 (26.2)
≥5	4,130 (4.0)
*Smoking status*	
None	65,905 (63.6)
Former	10,112 (9.8)
Current	27,602 (26.6)
*Regular physical activity (per week)*	
None	55,802 (53.9)
1–4	38,348 (37.0)
≥5	9,469 (9.1)
*Comorbidities*	
Hypertension	28,272 (27.3)
Diabetes mellitus	11,677 (11.3)
Dyslipidemia	17,901 (17.3)
Atrial fibrillation	277 (0.3)
Renal disease	782 (0.8)
*Oral health status*	
Number of missing teeth	
0	78,244 (75.5)
1–7	22,723 (21.9)
8–14	1,560 (1.5)
≥15	1,092 (1.1)
Number of dental caries	
0	82,846 (80.0)
1–5	19,797 (19.1)
≥6	976 (0.9)
*Oral hygiene behaviors*	
Frequency of tooth brushing (times/per day)	
0–1	16,569 (16.0)
2	53,611 (51.7)
≥3	33,439 (32.3)
Dental visit for any reason within the previous year	
No	61,159 (59.0)
Yes	42,460 (41.0)
Dental scaling within the previous year	
No	80,971 (78.1)
Yes	22,648 (21.9)

T: Tertile. Data are expressed as the mean ± standard deviation, or *n* (%).

**FIGURE 2:**
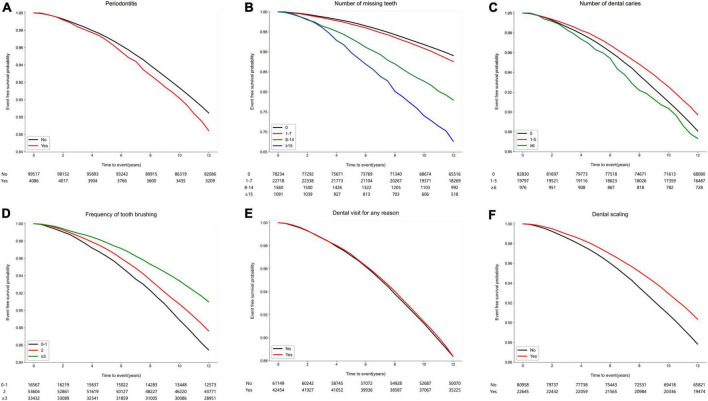
Kaplan–Meier survival curves free from cataract according to oral health status and oral hygiene behaviors. **(A)** Periodontitis (*p* < 0.001). **(B)** Number of missing teeth (*p* < 0.001). **(C)** Number of dental caries (*p* < 0.001). **(D)** Frequency of tooth brushing (times/per day) (*p* < 0.001). **(E)** Dental visit for any reason within the previous year (*p* = 0.739). **(F)** Dental scaling within the previous year (*p* < 0.001).

During the median follow-up of 12.2 years (interquartile range of 12.1–12.5 years), cataract developed in 12,114 (11.7%) participants. Figure 2 shows the Kaplan–Meier survival curves free from cataract according to oral health status and oral hygiene behaviors. The risk for incident cataract was higher when the participants had periodontitis and a higher number of missing teeth (*p* < 0.001). Better oral hygiene behaviors, namely, increased frequency of daily tooth brushing and a history of dental scaling within the previous year, were also associated with a reduced occurrence of cataract (*p* < 0.001). However, a history of dental visit for any reason was not a factor that increased or decreased the risk for cataract (*p* = 0.739).

Although periodontitis was positively correlated with the occurrence of incident cataract in univariable analysis (Supplementary Table 3), the significance of this correlation decreased in multivariable analysis (adjusted HR = 1.08, 95% CI = 0.99–1.17, *p* = 0.088) (Table 2). The number of missing teeth was associated with an increased risk for cataract occurrence. The following adjusted HRs (in reference to the subject with no missing teeth) were obtained: 1.31 (95% CI = 1.17–1.47, *p* < 0.001) for participants with 8–14 missing teeth, and 1.74 (95% CI = 1.55–1.96, *p* < 0.001) for participants with more than 15 missing teeth. Furthermore, the increased frequency of tooth brushing was negatively correlated with cataract occurrence. In reference to the participants who brushed their teeth less than once a day, the subjects who brushed their teeth twice a day (adjusted HR = 0.94, 95% CI = 0.89–0.98, *p* = 0.008) and more than thrice a day (adjusted HR = 0.84, 95% CI = 0.79–0.88, *p* < 0.001) had a decreased risk for incident cataract. Moreover, those who received dental scaling within one year showed a significantly lower risk for cataract (adjusted HR = 0.90, 95% CI = 0.86–0.94, *p* < 0.001). In contrast, the number of dental caries was not a factor associated with the increased risk for cataract. In addition, dental visit history for any reason was not a factor reducing the risk for cataract.

**TABLE 2:** The risk for occurrence of cataract according to oral health status and oral hygiene behaviors.

	Events, *n*	Person-years	Incidence rate per 1,000 person-years	Adjusted HR* (95% CI)	*P*-value
*Oral health status*					
Periodontitis					
No	11,558	1,138,096.07	10.16	1 (reference)	
Yes	556	45,938.58	12.10	1.08 (0.99, 1.17)	0.088
Number of missing teeth					
0	8,673	9,00,641.58	9.63	1 (reference)	
1–7	2,814	2,57,344.83	10.94	0.99 (0.94, 1.03)	0.479
8–14	324	16,029.06	20.21	**1.31** **(1.17, 1.47)**	**< 0.001**
≥15	303	10,019.17	30.24	**1.74** **(1.55, 1.96)**	**< 0.001**
Number of dental caries					
0	9,950	9,45,844.00	10.52	1 (reference)	
1–5	2,048	2,27,571.38	9.00	**0.89** **(0.85, 0.93)**	**< 0.001**
≥6	116	10,619.27	10.92	1.08 (0.90, 1.29)	0.424
*Oral hygiene behaviors*					
Frequency of tooth brushing (times/per day)					
0–1	2,362	1,83,128.42	12.90	1 (reference)	
2	6,693	6,11,158.26	10.95	**0.94** **(0.89, 0.98)**	**0.008**
≥3	3,059	3,89,747.97	7.85	**0.84** **(0.79, 0.88)**	**< 0.001**
Dental visit for any reason within the previous year					
No	7,141	6,96,666.72	10.25	1 (reference)	
Yes	4,973	4,87,367.92	10.20	1.01 (0.97, 1.04)	0.765
Dental scaling within the previous year					
No	9,878	9,20,311.47	10.73	1 (reference)	
Yes	2,236	2,63,723.18	8.48	**0.90** **(0.86, 0.94)**	**< 0.001**

*Adjusted for age, sex, body mass index, household income, alcohol consumption, smoking status, regular physical activity, comorbidities (hypertension, diabetes mellitus, dyslipidemia, atrial fibrillation, and renal disease). HR, hazard ratio; CI, confidence interval. Bold indicates statistically significant difference by Cox’s proportional hazards regression analysis.

## Discussion

The key findings of our study demonstrated that poor oral health status such as the presence of periodontitis and an increased number of missing teeth was associated with an increased risk of cataract occurrence. Conversely, better oral hygiene behaviors such as higher frequency of tooth brushing and a history of dental scaling were associated with a decreased risk of cataract occurrence. Recent evidence has indicated that chronic oral inflammation and infection lead to systemic inflammatory consequences and are associated with various systemic diseases. Periodontitis, a common oral inflammatory disease, increases the risk for cardiovascular diseases and diabetes (18–20). Tooth loss, an indicator of poor oral health status, is also associated with the increased risk for cardiovascular diseases (17, 21) and hypertension (22, 23). Conversely, an increased frequency of tooth brushing, a behavior that lowers oral inflammation, reduces the risk for stroke, (7) atrial fibrillation, and heart failure (1). In contrast to these reports, studies have rarely reported the association of oral health with cataract. Previous studies also suggested the potential relationship between periodontitis and eye diseases (24, 25). The development and progression of ocular diseases, such as glaucoma (26), age-related macular degeneration (27), and diabetic retinopathy (28), are likely affected by periodontitis. Moreover, limited evidence supports the effect of periodontitis or oral hygiene on cataract occurrence. In a retrospective study that evaluated the risk for cataract in subjects with and without periodontitis by referring to the national health insurance research database of Taiwan, subjects with periodontitis have a higher risk for cataract development than those without periodontitis (29). Considering these previous and our research results, our findings were meaningful because they showed that tooth loss, frequent tooth brushing, and dental scaling were associated with the risk of cataract occurrence. Furthermore, our results suggested that the control of periodontitis and chronic oral inflammation through prophylactic dental scaling and good oral hygiene behaviors could reduce the risk of future cataract occurrence.

Although the direct causal relationship between poor oral health and cataract occurrence could not be determined in our study, the following hypotheses might explain the association. In poor oral hygiene or oral disease status, a systemic inflammatory reaction induced by oral inflammatory conditions may elicit the excessive production of reactive oxygen species, such as superoxide, hydroxyl anions, and hydrogen peroxide in oral or periodontal tissues, as supported by clinical and animal studies (30–33). These reactive oxygen species can diffuse into the bloodstream (33, 34). This condition can cause the oxidation of various molecules in the blood; in turn, it can lead to circulating oxidative stress, which can progressively damage other organs (35, 36). Oxidative stress is an important factor in cataractogenesis in experimental animals and cultured lens models (37, 38). Therefore, increased oxidative stress levels due to chronic oral inflammation may negatively affect cataract development.

In our study, periodontitis showed positive tendency for association with cataract in multivariable analysis, although there was an association in univariable analysis. This may be because the association of other factors, i.e. vascular risk factors, with cataract development was greater than that of periodontitis. The results may be attributed to the nature of the included population, such as general population or ethnicity. Moreover, periodontitis was defined using the frequently used definition in most studies. Although this definition has a high diagnostic accuracy for periodontitis, periodontitis may have been already treated because treatment-related claim codes are considered; therefore, the risk for cataract may be relatively low. Nevertheless, our findings were meaningful because they suggested the possible link between periodontitis and the risk of cataract occurrence.

In the present study, the increased number of dental caries was not a factor associated with the increased risk of cataract. Since dental caries is a biofilm-mediated disease similar to periodontitis, a local inflammatory response through dental caries likely results in systemic inflammation via mechanisms similar to periodontitis (39, 40). However, dental caries unlikely triggers inflammatory responses of surrounding tissues until bacteria penetrate the root canal system of the teeth. Only advanced dental caries causes apical periodontitis, which is characterized by periodontal tissue destruction. Thus, the number of dental caries, including advanced and incipient ones, unlikely affects the increased risk of cataract.

This study has several limitations. First, residual confounding factors might exist and affect cataract development. Ultraviolet sunlight exposure, known to be positively correlated with cataracts (41, 42), was not included in the analyses because of the lack of information on sunlight exposure in the cohort database. Second, results of other races/ethnics might vary because study subjects only included Koreans. Third, no information of the detailed attachment loss was found in the NIHS-HEALS cohort database, and severity of periodontitis could not be investigated. Fourth, response bias such as social–desirability bias might occur because oral health behaviors were based on the self-reported questionnaire. However, this study has the following strengths. Large-scale long-term-tracked nationally representative data were used to elucidate the effect of oral health status and oral hygiene behaviors on cataract occurrence. Our results provided significant evidence supporting the benefits of maintaining good oral health for cataract prevention.

## Conclusion

Periodontitis and increased number of missing teeth may be associated with the increased risk of cataract. However, maintaining good oral hygiene through tooth brushing and dental scaling may reduce the risk of future cataract occurrence. Further studies should be performed to confirm the association between chronic oral inflammation and cataract.

## Data availability statement

The datasets presented in this article are not readily available because the data used in this study are available in the National Health Insurance Service-National Health Screening Cohort (NHIS-HEALS) database, but restrictions apply to the public availability of these data used under the license for the current study. Requests for access to the NHIS data can be made through the National Health Insurance Sharing Service homepage (http://nhiss.nhis.or.kr/bd/ab/bdaba021eng.do). For access to the database, a completed application form, research proposal, and application for approval from the institutional review board should be submitted to the inquiry committee of research support in the NHIS for review. Requests to access the datasets should be directed to http://nhiss.nhis.or.kr/bd/ab/bdaba021eng.do.

## Ethics statement

This study was approved by the human subjects Ethics Board of Ewha Womans University College of Medicine (2020-08-018). Written informed consent for participation was not required for this study in accordance with the national legislation and the institutional requirements.

## Author contributions

J-HP contributed to data interpretation and drafted the manuscript. HL and J-WK contributed to data analysis and interpretation. T-JS contributed to conception, design, data acquisition, interpretation, and critically revised the manuscript. All authors gave final approval and agreed to be accountable for all aspects of the work.
