# Supplementary material for: Better oral hygiene is associated with a reduced risk of cataract: A nationwide cohort study
Source: Front Med (Lausanne). 2023 Jan 3;9:1036785. doi: 10.3389/fmed.2022.1036785 (PMC9842665; doi:10.3389/fmed.2022.1036785)
Supplement: Supplementary file 1 [file Data_Sheet_1.docx]

**Supplemental Table 1**. Treatment Codes Related to Periodontitis.

| Code | Procedure |
| --- | --- |
| U2232 | Removal of dental calculus (one third of dental arch) |
| U2240 | Root planing |
| U2233 | Removal of dental calculus (whole dental arch) |
| U2221 | Dressing after periodontal treatment (removal of dental calculus, root planning, or curettage) |
| U1010 | Curettage (one third of dental arch) |
| U2211 | Dressing after dental surgery (simple) |
| U4454 | Incision and drainage (gingival abscess or pericoronal abscess) |
| U2222 | Dressing after periodontal treatment (other than removal of dental calculus, root planning, or curettage) |
| U0010 | Simple dressing |
| U1051 | Periodontal flap operation (simple) |
| U1052 | Periodontal flap operation (complicated) |
| U4455 | Incision and drainage (periodontal abscess or palatal abscess) |
| U1060 | Root conditioning |
| U2231 | Oral prophylaxis |
| M0111 | Simple dressing of wound |
| U4660 | Operculectomy |

**Supplemental Table 2**. Baseline characteristics of the study population according to the presence of periodontitis.

|  | **Total** | **Periodontitis (-)** | **Periodontitis (+)** | ***p* value** |
| --- | --- | --- | --- | --- |
| **No. of patients (%)** | 103619 | 99533 (96.1) | 4086 (3.9) |  |
| **Age** |  |  |  |  |
| Mean (year) | 51.64±8.74 | 51.58±8.73 | 53.12±8.98 | <.001 |
| **Sex** |  |  |  | <.001 |
| Male | 62248 (60.1) | 59530 (59.8) | 2718 (66.5) |  |
| Female | 41371 (39.9) | 40003 (40.2) | 1368 (33.5) |  |
| **Body mass index (kg/m2)** | 23.94±2.91 | 23.93±2.91 | 23.95±3.01 | 0.819 |
| **Household income** |  |  |  | <.001 |
| T1, lowest | 30353 (29.3) | 29116 (29.3) | 1237 (30.3) |  |
| T2 | 38001 (36.7) | 36334 (36.5) | 1667 (40.8) |  |
| T3, highest | 35265 (34.0) | 34083 (34.2) | 1182 (28.9) |  |
| **Alcohol consumption (per week)** |  |  |  | <.001 |
| None | 72370 (69.8) | 69764 (70.1) | 2606 (63.8) |  |
| 1-4 | 27119 (26.2) | 25878 (26.0) | 1241 (30.4) |  |
| ≥5 | 4130 (4.0) | 3891 (3.9) | 239 (5.9) |  |
| **Smoking status** |  |  |  | <.001 |
| None | 65905 (63.6) | 63550 (63.9) | 2355 (57.6) |  |
| Former | 10112 (9.8) | 9670 (9.7) | 442 (10.8) |  |
| Current | 27602 (26.6) | 26313 (26.4) | 1289 (31.6) |  |
| **Regular physical activity (per week)** |  |  |  | 0.209 |
| None | 55802 (53.9) | 53550 (53.8) | 2252 (55.1) |  |
| 1-4 | 38348 (37.0) | 36888 (37.1) | 1460 (35.7) |  |
| ≥5 | 9469 (9.1) | 9095 (9.1) | 374 (9.2) |  |
| **Comorbidities** |  |  |  |  |
| Hypertension | 28272 (27.3) | 26982 (27.1) | 1290 (31.6) | <.001 |
| Diabetes mellitus | 11677 (11.3) | 11094 (11.2) | 583 (14.3) | <.001 |
| Dyslipidemia | 17901 (17.3) | 17195 (17.3) | 706 (17.3) | 0.996 |
| Atrial fibrillation | 277 (0.3) | 271 (0.3) | 6 (0.2) | 0.128 |
| Renal disease | 782 (0.8) | 753 (0.8) | 29 (0.7) | 0.735 |
| **Oral health status** |  |  |  |  |
| Number of missing teeth |  |  |  | <.001 |
| 0 | 78244 (75.5) | 75651 (76.0) | 2593 (63.5) |  |
| 1-7 | 22723 (21.9) | 21374 (21.5) | 1349 (33.0) |  |
| 8-14 | 1560 (1.5) | 1450 (1.5) | 110 (2.7) |  |
| ≥15 | 1092 (1.1) | 1058 (1.1) | 34 (0.8) |  |
| Number of dental caries |  |  |  | <.001 |
| 0 | 82846 (80.0) | 79816 (80.2) | 3030 (74.2) |  |
| 1-5 | 19797 (19.1) | 18814 (18.9) | 983 (24.1) |  |
| ≥6 | 976 (0.9) | 903 (0.9) | 73 (1.8) |  |
| **Oral hygiene behaviors** |  |  |  |  |
| Frequency of tooth brushing (times/per day) |  |  |  | <.001 |
| 0-1 | 16569 (16.0) | 15814 (15.9) | 755 (18.5) |  |
| 2 | 53611 (51.7) | 51478 (51.7) | 2133 (52.2) |  |
| ≥3 | 33439 (32.3) | 32241 (32.4) | 1198 (29.3) |  |
| Dental visit for any reason within the previous year |  |  |  | 0.982 |
| No | 61159 (59.0) | 58748 (59.0) | 2411 (59.0) |  |
| Yes | 42460 (41.0) | 40785 (41.0) | 1675 (41.0) |  |
| Dental scaling within the previous year |  |  |  | <.001 |
| No | 80971 (78.1) | 77678 (78.0) | 3293 (80.6) |  |
| Yes | 22648 (21.9) | 21855 (22.0) | 793 (19.4) |  |

T: Tertile.

*p* value by Chi-square test for categorical variables and independent t-test for continuous variables. Data are expressed as the mean ± standard deviation, or n (%).

**Supplemental Table 3**. Risk factors for the occurrence of cataract.

|  | **Crude HR**  **(95% CI)** | **p-value** | **Adjusted HR***  **(95% CI)** | ***p* value** |
| --- | --- | --- | --- | --- |
| **Age** |  |  |  |  |
| <50 | 1 (reference) |  | 1 (reference) |  |
| ≥ 50 | **6.19 (5.89, 6.50)** | **<.001** | **5.31 (5.05, 5.58)** | **<.001** |
| **Sex** |  |  |  |  |
| Male | 1 (reference) |  | 1 (reference) |  |
| Female | **1.49 (1.44, 1.55)** | **<.001** | **1.34 (1.28, 1.40)** | **<.001** |
| **Body mass index (kg/m2)** | **1.03 (1.02, 1.03)** | **<.001** | 1.00 (0.99, 1.00) | 0.275 |
| **Household income** |  |  |  |  |
| T1, lowest | 1 (reference) |  | 1 (reference) |  |
| T2 | **0.85 (0.81, 0.88)** | **<.001** | 1.00 (0.96, 1.05) | 0.869 |
| T3, highest | **0.69 (0.66, 0.73)** | **<.001** | 0.96 (0.92, 1.00) | 0.070 |
| **Alcohol consumption (per week)** | ` |  |  |  |
| None | 1 (reference) |  | 1 (reference) |  |
| 1-4 | **0.69 (0.66, 0.72)** | **<.001** | **0.94 (0.90, 0.99)** | **0.017** |
| ≥5 | **1.35 (1.25, 1.47)** | **<.001** | **1.27 (1.17, 1.38)** | **<.001** |
| **Smoking status** |  |  |  |  |
| None | 1 (reference) |  | 1 (reference) |  |
| Former | **0.80 (0.75, 0.85)** | **<.001** | **1.14 (1.06, 1.22)** | **<.001** |
| Current | **0.70 (0.67, 0.73)** | **<.001** | 1.03 (0.98, 1.09) | 0.266 |
| **Regular physical activity (per week)** |  |  |  |  |
| None | 1 (reference) |  | 1 (reference) |  |
| 1-4 | **0.68 (0.65, 0.70)** | **<.001** | **0.84 (0.80, 0.87)** | **<.001** |
| ≥5 | **1.14 (1.08, 1.21)** | **<.001** | 1.04 (0.98, 1.10) | 0.199 |
| **Comorbidities** |  |  |  |  |
| Hypertension | **1.92 (1.86, 2.00)** | **<.001** | **1.32 (1.27, 1.38)** | **<.001** |
| Diabetes mellitus | **2.10 (2.01, 2.20)** | **<.001** | **1.57 (1.49, 1.64)** | **<.001** |
| Dyslipidemia | **1.52 (1.46, 1.59)** | **<.001** | **1.10 (1.05, 1.15)** | **<.001** |
| Atrial fibrillation | **1.96 (1.49, 2.57)** | **<.001** | 1.15 (0.87, 1.51) | 0.329 |
| Renal disease | **2.53 (2.19, 2.91)** | **<.001** | **1.35 (1.17, 1.56)** | **<.001** |
| **Oral health status** |  |  |  |  |
| Periodontitis |  |  |  |  |
| No | 1 (reference) |  | 1 (reference) |  |
| Yes | **1.20 (1.10, 1.31)** | **<.001** | 1.08 (0.99, 1.17) | 0.088 |
| Number of missing teeth |  |  |  |  |
| 0 | 1 (reference) |  | 1 (reference) |  |
| 1-7 | **1.14 (1.09, 1.19)** | **<.001** | 0.99 (0.94, 1.03) | 0.479 |
| 8-14 | **2.18 (1.95, 2.43)** | **<.001** | **1.31 (1.17, 1.47)** | **<.001** |
| ≥15 | **3.39 (3.02, 3.80)** | **<.001** | **1.74 (1.55, 1.96)** | **<.001** |
| Number of dental caries |  |  |  |  |
| 0 | 1 (reference) |  | 1 (reference) |  |
| 1-5 | **0.85 (0.81, 0.90)** | **<.001** | **0.89 (0.85, 0.93)** | **<.001** |
| ≥6 | 1.05 (0.88, 1.27) | 0.579 | 1.08 (0.90, 1.29) | 0.424 |
| **Oral hygiene behaviors** |  |  |  |  |
| Frequency of tooth brushing (times/per day) |  |  |  |  |
| 0-1 | 1 (reference) |  | 1 (reference) |  |
| 2 | **0.84 (0.80, 0.88)** | **<.001** | **0.94 (0.89, 0.98)** | **0.008** |
| ≥3 | **0.60 (0.57, 0.63)** | **<.001** | **0.84 (0.79, 0.88)** | **<.001** |
| Dental visit for any reason within the previous year |  |  |  |  |
| No | 1 (reference) |  | 1 (reference) |  |
| Yes | 0.99 (0.96, 1.03) | 0.739 | 1.01 (0.97, 1.04) | 0.765 |
| Dental scaling within the previous year |  |  |  |  |
| No | 1 (reference) |  | 1 (reference) |  |
| Yes | **0.78 (0.75, 0.82)** | **<.001** | **0.90 (0.86, 0.94)** | **<.001** |

^*^Adjusted for age, sex, body mass index, household income, alcohol consumption, smoking status, regular physical activity, comorbidities (hypertension, diabetes mellitus, dyslipidemia, atrial fibrillation, and renal disease).

HR = hazard ratio; CI = confidence interval.

Bold indicates statistically significant difference by Cox’s proportional hazards regression analysis.
